# Role of Spatial Heterogeneity in Muscle-Invasive Bladder Cancer on Overall Survival and Immunotherapy Response

**DOI:** 10.3390/cancers18050875

**Published:** 2026-03-09

**Authors:** Arjun Venkatesh, Reynier D. Rodriguez Rosales, Jean-Pierre Kanumuambidi, Yudai Ishiyama, Mohammed Al-Toubat, Hunter Sceats, Thomas D. Metzner, Shelby Sparks, Nicole Murray, Mark Bandyk, K. C. Balaji

**Affiliations:** 1 Department of Urology, University of Florida Jacksonville, Jacksonville, FL 32209, USA; avenkat1@sgu.edu (A.V.);; 2 School of Medicine, St. George’s University, St. George P.O. Box 7, Grenada; 3 Huntsman Cancer Institute, University of Utah, Salt Lake City, UT 84112, USA

**Keywords:** bladder cancer, location, genetic alteration, synthetic lethality, immune checkpoint inhibitors

## Abstract

Purpose: Tumor location influences survival in bladder cancer, potentially due to genetic heterogeneity driven by distinct embryological origins and structural compositions. We investigate location-specific somatic gene alterations (GAs) and their potential clinical implications in muscle-invasive bladder cancer (MIBC). Methods: We explored the role of the intra-bladder tumor location in determining survival and underlying genetic alterations in MIBC patients using multiple large independent databases. We analyzed the tumor location’s impact on survival using the Surveillance, Epidemiology, and End Results (SEER) database and validated these findings using cBioPortal (CBP), which also contains gene sequencing data, enabling a comparison of GA frequency by tumor location. We investigated GA combinations to identify potential synthetic lethal (SL) combinations and co-occurrence signatures for survival prediction. Using the ROC Plotter database, we explored how significantly altered genes affect the response to immune checkpoint inhibitors (ICI). Results: An analysis of 6712 SEER and 570 CBP patients revealed significant (*p* < 0.001) differences in overall survival stratified by tumor location, with trigone tumors showing the worst survival. Genomic analysis identified 35 genes with location-specific alteration frequencies. Three of these genes, CDKN2A, SPTAN1, and BIRC6, were significantly predictive of ICI response, and three genes were uniquely associated with a specific location: BPTF (anterior wall), RYR1, and OBSCN (dome). Furthermore, we identified 349 SL pairs from the 35 significantly altered genes, and a co-occurrence analysis revealed two novel gene pairs associated with improved survival. Conclusions: Intra-bladder tumor location determines survival and distinct genetic profiles in MIBC. These location-specific alterations predict ICI response and identify novel synthetic lethal targets, guiding precision oncology.

## Simple Summary

Bladder cancer is a complex disease, and our study demonstrates that the specific physical location of a tumor within the bladder (such as the dome or trigone) significantly impacts a patient’s survival and treatment response. By analyzing data from over 7000 patients across large national databases, we found that patients with tumors in the “trigone” region of the bladder had the lowest overall survival rates. Furthermore, we discovered that bladder cancers harbor distinct genetic mutations depending on their anatomical location. Importantly, we found that three of these location-specific altered genes can predict whether a patient is likely to benefit from immunotherapy. By mapping these regional genetic differences, we also identified novel genetic vulnerabilities—known as synthetic lethal combinations—that could be targeted with emerging drugs. Ultimately, considering exactly where a tumor is located inside the bladder can help doctors provide more personalized, effective therapies for patients with muscle-invasive bladder cancer.

## 1. Introduction

Bladder cancer is characterized by molecular, histologic, immune profile, and clinical heterogeneity. Previous studies in urothelial and adenocarcinoma of the bladder have shown that tumors located in the trigone and dome have worse pathology or survival compared to other sites, respectively [1,2]. This spatial variation may be explained by the bladder’s complex embryological development, where the trigone’s formation involves the integration of bladder smooth muscle and ureteral contributions [3,4,5], resulting in distinct gene expression profiles compared to other parts of the bladder [6]. Similar patterns of regional disease susceptibility due to embryological variations have been observed in other organ systems, suggesting that the bladder’s unique developmental origin could lead to region-specific differences in carcinogenesis and tumor behavior [7].

In this study, we aim to investigate the genetic alterations associated with tumor location and their influence on survival and response to immune checkpoint inhibitors (ICIs). By integrating large-scale genomic data with survival analyses, we identify distinct location-specific alteration patterns and demonstrate their association with differences in patient survival. Moreover, our findings reveal that several of these significantly altered genes predict the response to ICI, with potential clinical implications. This multifaceted analysis enhances our understanding of spatial heterogeneity in bladder cancer and its clinical implications.

## 2. Methods

**Figure 1:**
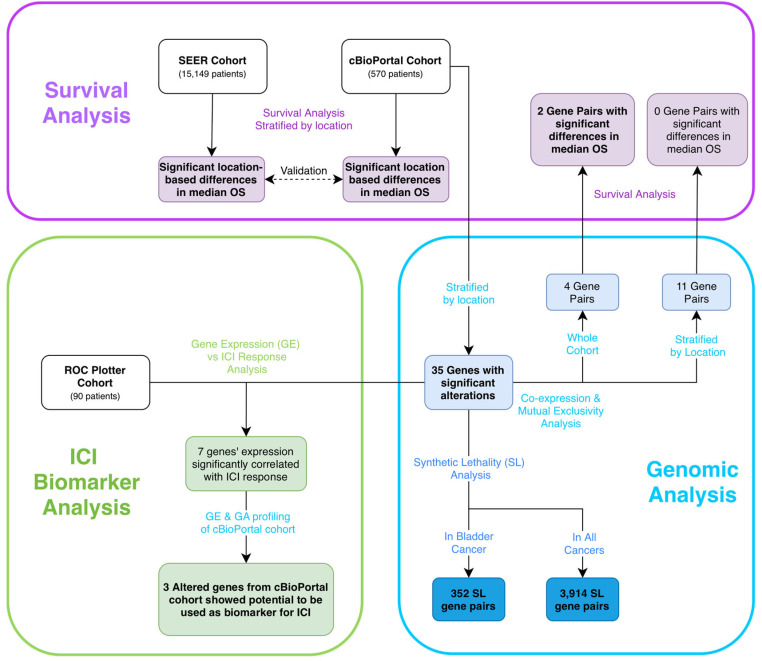
Study overview flowchart. Key outcomes indicated by bolded text. Abbreviations: OS—Overall Survival; SL—Synthetical Lethality; ICI—Immune Checkpoint Inhibitor; GE—Gene Expression; GA—Gene Alteration.

The study overview is outlined in Figure 1. This study protocol received ethical approval from the University of Florida IRB (Protocol # NH00046154).

### 2.1. Survival Analysis

Through the Surveillance, Epidemiology, and End Results (SEER) Program, which covers 48% of the US population, we used the SEER 17 database (2000–2021) to gather survival data. Patients with muscle-invasive bladder cancer staged as American Joint Committee on Cancer (AJCC) code T2 or higher were included in the analysis. Patients were stratified into cohorts based on International Classification of Diseases (ICD) codes by location: 67.0 (trigone), 67.1 (dome,) 67.2 (lateral wall), 67.3 (anterior wall), 67.4 (posterior wall), 67.5 (bladder neck), 67.6 (ureteric orifice), 67.7 (urachus), 67.8 (overlapping), and 67.9 (unspecified site). The number of patients was filtered to ensure at least 5 patients were in each ICD cohort. Demographic information collected included age, sex, race, and OS. TNM stage information was collected and categorized as T2, T3+ (T3 or higher), N+ (node positive), M0 (non-metastatic), or M1 (metastatic). Cystectomy status was collected as none, partial, simple, or radical. Kaplan–Meier survival curves were generated for each bladder region and log-rank test was used to compare survival distributions between cohorts. Hazard ratios (HRs) and 95% confidence intervals (CIs) were calculated using Cox proportional hazards regression models to assess the association between tumor location and survival outcomes, with or without adjustment for TNM staging and cystectomy status. *p*-values were corrected using the Benjamini–Hochberg (BH) method for multiple comparisons, with *p* < 0.05 considered significant. Statistical analyses were performed using R version 4.4.1 and RStudio version 2024.09.1+394 along with “survival” and “survminer” packages.

### 2.2. Genomic Analysis

The cBioPortal (CBP) for Cancer Genomics is an open platform which allows the exploration and analysis of multidimensional genomic data aggregated from publicly available datasets. All studies labeled as bladder urothelial carcinoma were identified, which were published from 2013 to 2022. Patients labeled as AJCC T2+ were then stratified by ICD code and filtered to ensure at least 5 patients were in each ICD cohort. A similar demographic data collection and survival analysis was performed, following the same approach described for the SEER cohort in the preceding paragraph. Detailed TNM data and cystectomy data were available only in less than a third of this cohort and, therefore, were not analyzed as independent covariates. Genetic alteration (GA) frequencies were compared using Chi-square tests, focusing on genes with an alteration frequency greater than 5% as per current conventions [8]. Genes found to be significantly altered underwent further post hoc pairwise analysis, comparing all location combinations for each gene to identify specific location(s) with significantly higher alteration frequencies.

Synthetic lethality refers to a genetic interaction where the simultaneous alteration of two genes leads to cell death, whereas an alteration in either gene alone is viable. To identify potential novel synthetic lethal (SL) combinations, the location-specific genes were subsequently queried in the Synthetic Lethality Online Analysis Database (SLOAD), a publicly available resource which utilizes machine learning to provide precise cancer-specific SL interactions [9]. Additionally, the location-specific genes underwent a co-occurrence and mutual exclusivity analysis within that location’s cohort, in addition to the entire cohort. Using Kaplan–Meier analysis, we compared the survival between the significant genes from the pairwise analysis that were combined with their co-occurring genes (log odds2 ratio ≥ 2) to create a unique gene signature. Cox proportional hazards regression models were used to assess the association between gene signature and survival outcomes, with or without adjustment for location, age, and sex.

### 2.3. Response to Immune Checkpoint Inhibitors

To explore how identified gene alterations affect response to immune checkpoint inhibitors (ICIs), we utilized the ROC Plotter database (rocplot.org) [10]. This database, which leverages transcriptome-level expression data, contains patients annotated as non-responders and responders, who are defined as those who experienced progression-free survival longer than 12 months, or had a partial or complete response. To evaluate the potential of the identified gene alterations as predictive biomarkers for response to immune checkpoint inhibitors, we used receiver operating characteristic (ROC) curves [11]. The area under the curve (AUC) of an ROC plot quantifies biomarker performance, with values ranging from 0.5 (no classification power) to 1 (perfect classification). Using the ROC Plotter tool, we analyzed 90 bladder cancer patients who underwent anti-programmed cell death protein 1, anti-programmed death-ligand 1, and/or anti-cytotoxic T-lymphocyte-associated protein 4 therapies to derive ROC *p*-values for each gene of interest.

For genes showing significant association (ROC *p*-value < 0.05), we performed detailed genetic alteration profiling within the CBP cohort to decipher potential differential expression effects driven by these GAs. This genetic profiling included comprehensive characterization of mutations (missense, truncating, splice, and fusion) and copy number variations (deep deletion and amplification) [12,13,14]. We predicted that GAs such as deep deletions and truncating and pathogenic missense mutations are likely to result in loss of gene expression, whereas other pathogenic variants may result in variable effects. To validate these predictions, we analyzed mRNA expression levels from the CBP cohort. Because these data came from four independent studies using different mRNA quantification methods, we analyzed each study’s expression data separately to avoid batch effects. To further understand the potential mechanisms underlying ICI response, we utilized TIMER2.0, an established computational tool that integrates six algorithms to estimate immune cell infiltration from transcriptomic data [6]. We analyzed the correlation between our genes of interest and various immune cell populations.

## 3. Results

### 3.1. Demographics

The patients’ demographic information is highlighted in Table 1. Out of 9,759,718 cases in the SEER 17 database, 15,149 patients were found to have AJCC T2+ bladder cancer: trigone (1042; 6.88%), dome (678; 4.48%), lateral wall (2676; 17.66%), anterior wall (522; 3.45%), posterior wall (1082; 7.14%), bladder neck (488; 3.22%), ureteric orifice (181; 1.19%), urachus (43; 0.28%), overlapping sites (3007; 19.85%), and unspecified sites (5430; 35.84%). Of these, 10,518 (69.43%) had T2 disease while 4631 (30.57%) had T3+ disease. The N stage distribution showed that 2864 (18.91%) were N+, and M1+ disease was present in 2055 (13.57%) patients. Regarding surgical management, 9923 (65.5%) patients did not undergo cystectomy, while 4756 (31.4%) received radical cystectomy, 334 (2.2%) underwent partial cystectomy, and 136 (0.9%) had simple cystectomy. Out of a total of 2980 patients with a diagnosis of bladder urothelial carcinoma on CBP, we included all 570 patients coded as AJCC Stage T2+ patients as our study cohort. This included patients with tumors in the trigone (84; 14.7%), dome (47; 8.25%), lateral wall (214; 37.5%), anterior wall (64; 11.2%), and posterior wall (161; 28.2%). The dataset included < five patients in the remaining locations and therefore, they were not included in the analysis.

### 3.2. Survival Analysis of SEER and cBioPortal

**Figure 2:**
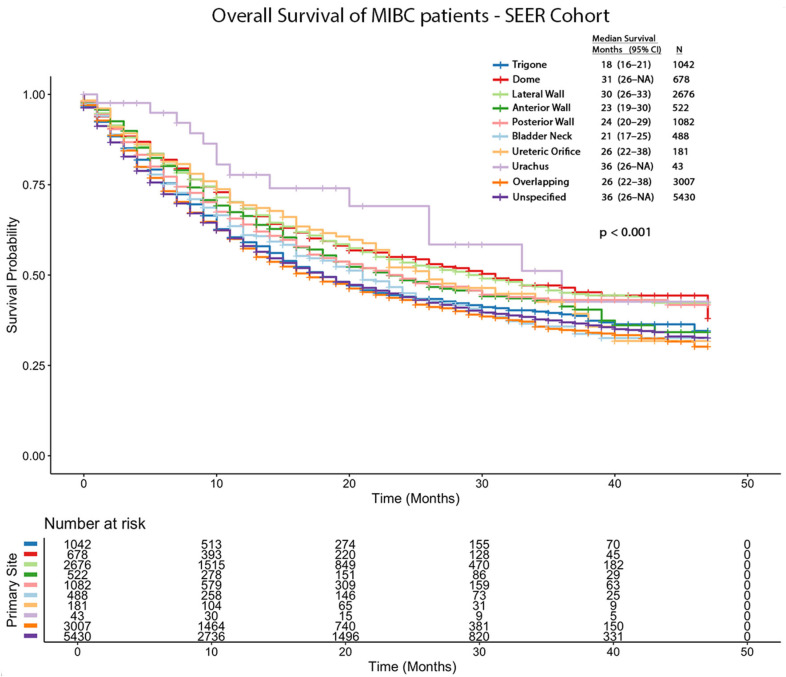
Kaplan–Meier curves showing overall survival of MIBC patients from SEER cohort stratified by tumor location.

The survival analysis of the SEER cohort revealed significant differences between tumor locations. The median OS in months and 95% CIs are as follows: trigone (18; 16–21), dome (31; 26-NA), lateral wall (30; 26–33), anterior wall (23; 19–30), posterior wall (24; 20–29), bladder neck (21; 17–25), ureteric orifice (26; 22–38), urachus (36; 26-NA), overlapping sites (17; 16–19), and unspecified sites (18; 17–19). After adjusting for TNM stage and surgical management, and using trigone tumors as the reference group, the following hazard ratios (HRs) were calculated: dome (HR = 0.86; 95% CI: 0.74–0.99; *p* = 0.04), lateral wall (HR = 0.83; 95% CI: 0.75–0.93; *p* < 0.001), anterior wall (HR = 0.92; 95% CI: 0.79–1.08; *p* = 0.306), posterior wall (HR = 0.92; 95% CI: 0.81–1.05; *p* = 0.202), bladder neck (HR = 0.87; 95% CI: 0.74–1.02; *p* = 0.078), ureteric orifice (HR = 0.86; 95% CI: 0.67–1.09; *p* = 0.200), urachus (HR = 0.47; 95% CI: 0.27–0.80; *p* = 0.005), overlapping sites (HR = 1.12; 95% CI: 1.01–1.24; *p* = 0.029), and unspecified sites (HR = 1.04; 95% CI: 0.94–1.14; *p* = 0.456). The overall survival Kaplan–Meier curves are shown in Figure 2.

**Figure 3:**
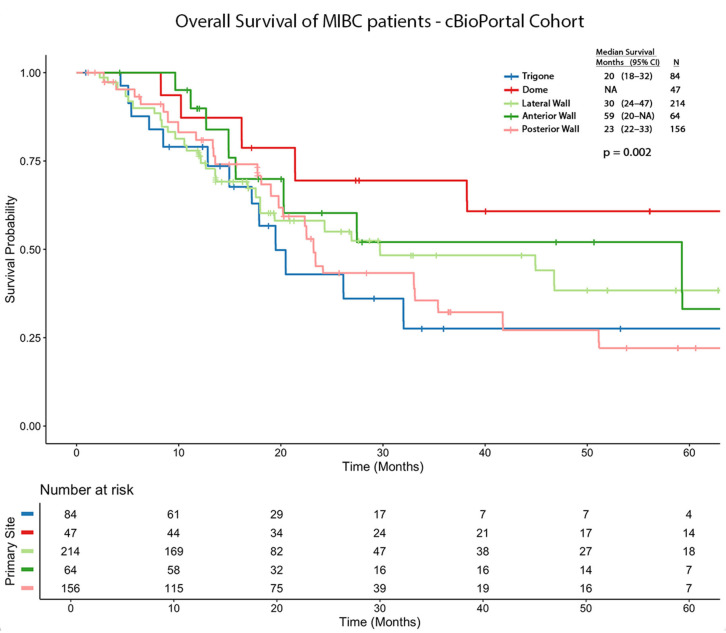
Kaplan–Meier curves showing overall survival of MIBC patients from cBioPortal cohort stratified by tumor location.

The survival analysis data was validated using an independent cBioPortal cohort. The median OS in months and 95% CIs are as follows: trigone (20; 18–32), dome (NA), lateral wall (30; 24–47), anterior wall (59; 20-NA), and posterior wall (23; 22–33). Using trigone tumors as the reference group, significant survival differences emerged across tumor locations, yielding the following hazard ratios (HRs): dome (HR = 0.364; 95% CI: 0.209–0.634; *p* < 0.001), lateral wall (HR = 0.733; 95% CI: 0.519–1.035; *p* = 0.078), anterior wall (HR = 0.589; 95% CI: 0.367–0.944; *p* = 0.027), and posterior wall (HR = 0.880; 95% CI: 0.618–1.25; *p* = 0.477). The overall survival Kaplan–Meier curves are shown in Figure 3.

### 3.3. CBP Genomic Analysis

Of the total 14,154 genes reported, 423 (3%) genes were altered in more than 5% of the cohort. While the overall proportion of patients harboring any genetic alteration did not significantly differ across bladder locations, the gene-specific Chi-square analysis revealed 35 distinct genes (*p* < 0.01) with alteration frequencies that varied significantly by location (Table 2). Of these, 10, 14, 4, and 7 genes were significantly altered in the anterior wall, dome, posterior wall, and trigone, respectively, with none in the lateral wall. The pairwise comparison of these genes revealed three genes which were significantly more frequently altered in one site compared to all other sites: BPTF (anterior wall), OBSCN (dome), and RYR1 (dome) (Table 2). To explore synthetic lethal interactions, we queried these three genes in SLOAD [9]. In bladder cancer, RYR1 had two candidate SL partners (METTL4, NDUFV2) and 85 combinations of synthetic lethal partners across all cancers; OBSCN had none in bladder cancer and 51 across all cancers; and BPTF had one (TRIM3) in bladder cancer and 116 across all cancers (Supplementary Table S1). The analysis of the remaining 32 significantly mutated genes revealed 349 and 3662 predicted SL pairs in bladder cancer and across all cancers, respectively, with only 2.36% verified to date.

**Figure 4:**
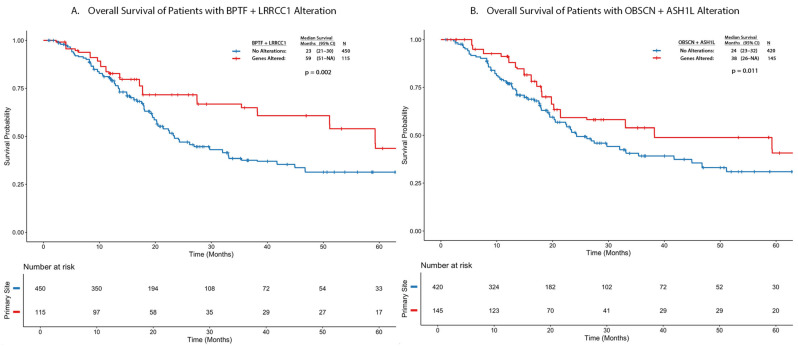
Kaplan–Meier curves showing overall survival of MIBC patients from cBioPortal cohort (**A**) with BPTF + LRRCC1 gene signature; (**B**) with OBSCN + ASH1L gene signature.

Next, we carried out a co-occurrence and mutual exclusivity analysis of the 3 genes (BPTF, OBSCN, RYR1) with the 35 significantly mutated genes by location (Supplementary Table S2). While there were no genes that were significantly co-occurring or mutually exclusive with BPTF, there were six and five genes co-occurring with OBSCN and RYR1, respectively, in the dome, with no mutual exclusivity. The OS was not significantly affected by BPTF, OBSCN, or RYR1 individually, or by their respective co-occurring gene signatures in the dome. Furthermore, we carried out a co-occurrence and mutual exclusivity analysis of the three genes, agnostic of location. We found 81 combinations with significant co-occurrences, and none were mutually exclusive. Of these, four gene combinations exhibited a log odds2 ratio (LOR) ≥ 2 (*p* < 0.001): BPTF/LRRCC1, OBSCN/ASH1L, RYR1/KMT2B, and RYR1/FCGBP (Supplementary Table S2). The survival analysis of the co-occurring gene signatures was not significant for RYR1 + KMT2B + FCGBP but was associated with significantly better survival compared to the genetically unaltered tumors in BPTF + LRRCC1 (HR = 0.532; 95% CI: 0.372–0.762; *p* < 0.001) and OBSCN + ASH1L (HR = 0.711; 95% CI: 0.530–0.953; *p* = 0.022) (Figure 4).

### 3.4. Response to Immune Checkpoint Inhibitors

**Figure 5:**
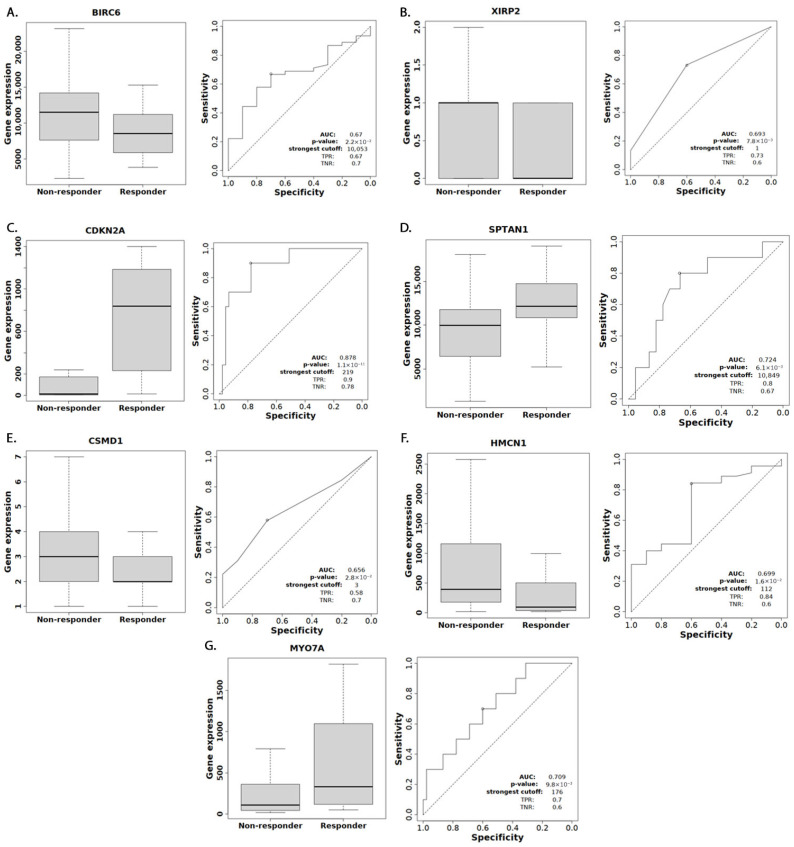
The receiver operating characteristic (ROC) curves for 7 out of 35 differentially altered genes by location, which significantly influenced the response to immune checkpoint inhibitors. These data are derived from the ROC Plotter online database: (**A**) BIRC6, (**B**) XIRP2, (**C**) CDKN2A, (**D**) SPTAN1, (**E**) CSMD1, (**F**) HMCN1, (**G**) MYO7A.

**Figure 6:**
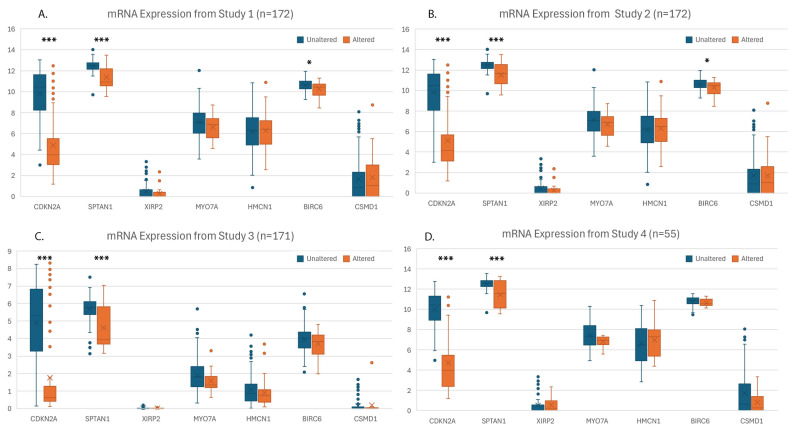
The mRNA expression profiles of the same genes from the cBioPortal cohort were analyzed. As this cohort is derived from multiple studies, each employing different mRNA quantification methods, the expression profiles of each individual study is depicted in (**A**–**D**).* = *p*-value < 0.01; *** = *p*-value < 0.001.

Using the ROC Plotter database, we evaluated the potential of specific genes as predictive biomarkers for the response to ICIs in bladder cancer. CDKN2A, SPTAN1, XIRP2, and MYO7A expression demonstrated significant associations with therapeutic response, with ROC *p*-values of <0.001, 0.006, 0.008, and 0.01, respectively, and false discovery rates (FDR) below 5%. HMCN1 (*p* = 0.016), BIRC6 (*p* = 0.022), and CSMD1 (*p* = 0.028) were also significant but their FDRs exceeded 5% after adjusting for multiple comparisons (Figure 5). Increased expression of CDKN2A, SPTAN1, and MYO7A, and decreased expression of XIRP2, HMCN1, BIRC6, and CSMD1 were associated with the response to ICIs. The GA characterization of the CBP cohort in these seven genes is shown in Supplementary Figure S1. Our mRNA expression analysis revealed CDKN2A and SPTAN1 to have significantly (*p* < 0.001) decreased expression across all four studies. BIRC6 was significantly decreased in two studies (*p* = 0.007/0.016) but not significantly in the other two. The remaining genes showed non-significant expression patterns (Figure 6). Finally, using TIMER2.0, we evaluated the immune cell infiltration patterns of the three significant genes. As shown in Supplementary Table S3, each gene demonstrated significant correlations with diverse immune populations, including CD8+, NK, and myeloid-derived suppressor cells, consistent with their potential roles in shaping the tumor microenvironment and influencing ICI outcomes.

## 4. Discussion

Both the SEER and CBP databases demonstrate significant differences in OS based on tumor location, with the trigone consistently associated with the poorest median OS: 18 (95% CI: 16–21) and 20 (95% CI: 18–32) months, respectively. These survival differences persist even after adjusting for staging and surgical management, suggesting the independent prognostic value of tumor location. This finding, consistent with a previous SEER analysis [2], may stem from several factors: the trigone’s unique embryological development involving bladder smooth muscle and ureteral integration [4,5], anatomical position facilitating prolonged carcinogen exposure [6], and potential diagnostic delays due to symptom misattribution [15]. These observations have important implications for clinical decision-making in MIBC management.

We offer a new lens through which to view the clinical management of MIBC, where tumor location could serve as an accessible and clinically relevant variable. The consistently poor prognosis associated with trigone tumors, even after adjusting for staging and surgical management, could inform adjuvant therapy decisions. In cases where the benefit of adjuvant immunotherapy is uncertain, trigone origin could be an additional factor favoring treatment. Furthermore, our identification of location-specific genetic alterations opens avenues for targeted therapies. While genes like RYR1, frequently altered in dome tumors, lack direct inhibitors, their synthetic lethal partners (METTL4, NDUFV2) represent novel therapeutic targets for this patient subgroup. Finally, tumor location may serve as a surrogate marker for immunotherapy response. The high frequency of CDKN2A alterations in anterior wall tumors suggests that these patients may be less likely to benefit from ICIs, given that CDKN2A loss is linked to immunotherapy resistance. This spatial–genomic information could help stratify patients for immunotherapy trials and guide biomarker development.

Our findings demonstrate spatially heterogeneous GAs across 35 identified genes in MIBC patients. Among these genes, three—RYR1, OBSCN, and BPTF—are uniquely and significantly altered in specific locations compared to all others. The bladder dome, which is associated with RYR1 and OBSCN alterations, possesses distinct structural and molecular characteristics that may contribute to its unique genetic landscape. It contains a higher smooth muscle content and acts as a pacemaker site for bladder contractions due to a greater concentration of interstitial cells of Cajal (ICCs) and elevated c-KIT expression [16,17]. Additionally, the presence of the urachus, a vestigial remnant of the allantois, at the dome introduces a unique microenvironment [18]. The anterior wall-associated BPTF alteration is interesting given that mechanical studies have shown that this region exhibits the least directional anisotropy compared to other bladder regions, suggesting unique structural properties that might influence tumor development [19].

The identification of these location-specific alterations provides potential insights into bladder cancer biology. RYR1, encoding ryanodine receptor 1, is integral to calcium signaling in urinary bladder smooth muscle and has been associated with higher tumor mutational burden and prognosis stratification in bladder cancer [20,21]. OBSCN, encoding obscurin, plays a role in cell adhesion and signaling, and while it has been implicated in breast and pancreatic cancers, its role in bladder cancer has not been previously described [22,23]. BPTF, a key player in chromatin remodeling, is particularly interesting as previous work has shown that circ-BPTF, a circular RNA from BPTF exons, is upregulated in bladder cancer tissues, correlating with higher tumor grade and poorer prognosis [24].

Our analysis identifies several SL interactions that could be exploited for targeted therapy development. These interactions are particularly interesting as METTL4, NDUFV2, and TRIM3 have demonstrated roles in various other cancers but remain unexplored in bladder cancer [25,26,27]. BPTF inhibitors like BZ1 and AU1 show promise in cancer therapy by targeting SL, with preclinical studies highlighting their potential in combination treatments [28]. Beyond synthetic lethality, we also explore gene co-occurrence patterns. Our analysis across the entire cohort identified two gene signatures (BPTF + LRRCC1 and OBSCN + ASH1L) associated with significantly improved survival compared to genetically unaltered tumors.

Immunotherapy, particularly ICIs, has shifted the treatment paradigm in bladder cancer management. Although ICI response data is often derived from cohorts with metastatic disease, genomic profiling of the primary tumor is a cornerstone of modern practice used to guide systemic therapy. Evolving biomarkers such as high tumor mutational burden, mutations in DNA damage response genes, and microsatellite instability are now used to inform treatment decisions [29,30]. More importantly, immunotherapy is an established option in the adjuvant (non-metastatic) setting for high-risk MIBC after cystectomy, making the identification of predictive biomarkers in resected primary tumors a critical clinical need [6]. Our analysis identified that higher expression of CDKN2A, SPTAN1, and MYO7A and lower expression of XIRP2, HMCN1, BIRC6, and CSMD1 are associated with improved immunotherapy response. CDKN2A, a well-established tumor suppressor gene associated with bladder cancer progression [31,32,33], shows substantial deep deletions in our genetic characterization, which is corroborated by significantly decreased mRNA expression across all four studies in our cohort. Given that the ROC Plotter data indicates that higher CDKN2A expression correlates with ICI response, these findings suggest that MIBC patients with CDKN2A alterations may be less likely to benefit from ICI.

Our TIMER2.0 analysis confirms CDKN2A’s positive correlation with ICI response mediators (CD8+ T cells, dendritic cells, NK cells; *p* < 0.001), consistent with studies showing that CDKN2A loss predicts immunotherapy resistance [6,33,34,35,36]. SPTAN1, a cytoskeletal protein that modulates tumor microenvironment in colorectal cancer [37], shows decreased expression across all studies and correlates with dendritic and NK cells, explaining its association with ICI responsiveness. BIRC6, a protein involved in inhibiting apoptosis [38], displays variable expression and contradictory immune correlations, with MIBC patients harboring BIRC6 alterations potentially responding better to immunotherapy despite associations with immunosuppressive cells. The remaining genes show mixed or non-significant expression patterns, making their clinical implications less clear.

Our study has several limitations, including our focus on MIBC only, potential misclassification from ICD codes, the large proportion of patients with ‘overlapping’ or ‘unspecified’ tumor locations, the lack of granularity regarding surgical quality (e.g., number of lymph nodes examined), specific peri-operative systemic therapies, and challenges with integrating samples from multiple studies. Additionally, the macro-level observational nature of the study precludes causal inferences. Future high-resolution spatial transcriptomics and functional studies are necessary to validate the biological impact of the identified altered genes and their role in tumor behavior.

## 5. Conclusions

Our findings demonstrate that spatial genetic heterogeneity in bladder cancer is associated with survival outcomes and response to ICI therapies. We identify significant region-specific genetic alterations, synthetic lethal interactions, and co-occurring gene signatures that provide potential therapeutic targets and prognostic indicators. Our integrated analysis uncovers novel predictive biomarkers for ICI response through correlations between genetic alterations, gene expression, and immune cell infiltration. These discoveries underscore the importance of considering tumor heterogeneity in precision medicine strategies, as location-based genetic profiles may guide treatment decisions and improve patient outcomes.

## Figures and Tables

**Table 1:** Demographic data of the SEER and cBioPortal cohorts.

Variable		Trigone	Dome	Lateral Wall	Anterior Wall	Posterior Wall	Bladder Neck	Ureteric Orifice	Urachus	Overlapping Sites	Unspecified Sites	*p*-Value
SEER Cohort												
**Total,***n* (%)		1042 (6.88)	678 (4.48)	2676 (17.66)	522 (3.45)	1082 (7.14)	488 (3.22)	181 (1.19)	43 (0.28)	3007 (19.85)	5430 (35.84)	
**T-Stage**												**<0.001**
	**T2**	763 (73.2)	468 (69.0)	2050 (76.6)	372 (71.3)	797 (73.7)	332 (68.0)	147 (81.2)	10 (23.3)	1892 (62.9)	3687 (67.9)	
	**T3+**	279 (26.8)	210 (31.0)	626 (23.4)	150 (28.7)	285 (26.3)	156 (32.0)	34 (18.8)	33 (76.7)	1115 (37.1)	1743 (32.1)	
**N-Stage**												**<0.001**
	**N0**	812 (77.9)	543 (80.1)	2194 (82.0)	412 (78.9)	844 (78.0)	382 (78.3)	140 (77.3)	31 (72.1)	2235 (74.3)	4010 (73.8)	
	**N1+**	178 (17.1)	102 (15.0)	398 (14.9)	91 (17.4)	210 (19.4)	78 (16.0)	34 (18.8)	7 (16.3)	678 (22.5)	1088 (20.0)	
	**NX**	52 (5.0)	33 (4.9)	84 (3.1)	19 (3.6)	28 (2.6)	28 (5.7)	7 (3.9)	5 (11.6)	94 (3.1)	332 (6.1)	
**M-Stage**												**<0.001**
	**M0**	887 (85.1)	599 (88.3)	2385 (89.1)	463 (88.7)	947 (87.5)	428 (87.7)	157 (86.7)	30 (69.8)	2611 (86.8)	4587 (84.5)	
	**M1**	155 (14.9)	79 (11.7)	291 (10.9)	59 (11.3)	135 (12.5)	60 (12.3)	24 (13.3)	13 (30.2)	396 (13.2)	843 (15.5)	
**Age Group;** median		75–79 yrs	75–79 yrs	70–74 yrs	70–74 yrs	70–74 yrs	75–79 yrs	75–79 yrs	55–59 yrs	70–74 yrs	70–74 yrs	
**Sex;** n (%)												**<0.001**
	**Male**	742 (71.2)	477 (70.4)	2003 (74.9)	389 (74.5)	795 (73.5)	404 (82.8)	133 (73.5)	27 (62.8)	2139 (71.1)	4061 (74.8)	
	**Female**	300 (28.8)	201 (29.6)	673 (25.1)	133 (25.5)	287 (26.5)	84 (17.2)	48 (26.5)	16 (37.2)	868 (28.9)	1369 (25.2)	
**Race**; n (%)												**<0.001**
	**White**	915 (87.81)	572 (84.37)	2376 (88.79)	437 (83.72)	960 (88.72)	422 (86.48)	157 (86.74)	24 (55.81)	2558 (85.1)	4621 (85.1)	
	**Black**	67 (6.43)	58 (8.55)	162 (6.05)	47 (9.00)	68 (6.28)	35 (7.17)	8 (4.42)	7 (16.28)	273 (9.08)	409 (7.53)	
	**Asian**	45 (4.32)	41 (6.05)	115 (4.30)	32 (6.13)	42 (3.88)	28 (5.74)	11 (6.08)	11 (25.58)	148 (4.92)	347 (6.39)	
	**American Indian**	8 (0.77)	4 (0.59)	14 (0.52)	1 (0.19)	5 (0.46)	3 (0.61)	3 (1.66)	1 (2.33)	11 (0.36)	18 (0.33)	
	**Unknown**	7 (0.67)	3 (0.44)	9 (0.34)	5 (0.96)	7 (0.65)	NA	2 (1.10)	NA	17 (0.57)	35 (0.64)	
**cBioPortal Cohort**												
**Total,** n (%)		84 (14.7)	47 (8.2)	214 (37.5)	64 (11.2)	161 (28.2)						
**Age;** median (IQR)		73 (66–78)	70 (56–74)	67 (60–73)	69 (63–79)	70 (61–76)	-	-	-	-	-	**<0.001**
**Sex;** n (%)												**0.006**
	**Male**	57 (67.9)	29 (61.7)	172 (80.37)	55 (85.9)	121 (75.2)	-	-	-	-	-	
	**Female**	27 (32.1)	18 (38.3)	42 (19.6)	9 (14.1)	40 (24.8%)	-	-	-	-	-	
**Race**; n (%)												0.068
	**White**	77 (91.7)	35 (74.5)	183 (85.5)	58 (90.6)	147 (91.3)	-	-	-	-	-	
	**Black**	3 (3.8)	6 (12.8)	16 (7.5)	3 (4.7)	14 (8.7)	-	-	-	-	-	
	**Asian**	0 (0)	3 (6.4)	9 (4.2)	3 (4.7)	0 (0)	-	-	-	-	-	
	**Unknown**	4 (4.8)	3 (6.4)	6 (2.8)	0 (0)	0 (0)	-	-	-	-	-	

Bolded values indicate a significant (*p* < 0.05) result.

**Table 2:** Chi-square and post hoc pairwise analysis of cBioPortal cohort comparing mutations across the five available bladder locations.

	Mutation Frequency						
Gene	Trigone	Dome	Lateral Wall	Anterior Wall	Posterior Wall	Highest Mutation Frequency	*p*-Value
BPTF	12%	12%	5%	34%	9%	Anterior Wall	**0.001**
MYO7A	13%	9%	5%	20%	6%	Anterior Wall	0.001
SPTAN1	12%	17%	13%	25%	6%	Anterior Wall	0.002
CDKN2A	33%	32%	35%	53%	50%	Anterior Wall	0.002
LRRCC1	17%	17%	9%	22%	6%	Anterior Wall	0.003
IGSF10	6%	7%	7%	19%	17%	Anterior Wall	0.004
AKAP9	6%	14%	12%	27%	12%	Anterior Wall	0.006
LAMA3	17%	7%	7%	20%	15%	Anterior Wall	0.008
ASH1L	13%	7%	14%	30%	14%	Anterior Wall	0.009
PTPRT	6%	21%	12%	22%	7%	Anterior Wall	0.01
KMT2A	25%	30%	8%	5%	14%	Dome	0.001
OBSCN	11%	38%	10%	15%	21%	Dome	**0.001**
TP53	60%	77%	53%	30%	54%	Dome	0.001
DNAH11	29%	34%	16%	5%	14%	Dome	0.001
SYNE1	24%	38%	18%	31%	12%	Dome	0.001
FCGBP	15%	26%	8%	5%	6%	Dome	0.001
BIRC6	23%	26%	13%	5%	7%	Dome	0.001
XIRP2	19%	28%	11%	5%	10%	Dome	0.001
RYR1	17%	38%	14%	17%	14%	Dome	**0.002**
HMCN1	26%	45%	20%	34%	22%	Dome	0.002
KMT2B	18%	24%	7%	19%	12%	Dome	0.003
EP300	15%	34%	23%	11%	14%	Dome	0.004
DNAH5	32%	43%	21%	15%	24%	Dome	0.004
MCM3AP	13%	21%	7%	5%	6%	Dome	0.007
F5	11%	19%	9%	17%	24%	Posterior Wall	0.001
SI	7%	19%	7%	13%	19%	Posterior Wall	0.002
SYNE2	17%	19%	8%	10%	20%	Posterior Wall	0.009
PIK3CA	25%	21%	19%	20%	35%	Posterior Wall	0.01
CSMD1	27%	15%	7%	13%	21%	Trigone	0.001
RELN	23%	14%	6%	18%	9%	Trigone	0.001
ADCY2	32%	15%	17%	7%	17%	Trigone	0.002
CEP350	20%	17%	6%	9%	14%	Trigone	0.005
NF1	20%	19%	8%	16%	8%	Trigone	0.006
ANK3	20%	11%	6%	11%	11%	Trigone	0.007
TENM3	20%	19%	10%	5%	7%	Trigone	0.01

Bolded values indicate a significant (*p* < 0.05) result.

## Data Availability

The original contributions presented in this study are included in the article/Supplementary Material. Further inquiries can be directed to the corresponding authors.

## Supplementary Materials

The following supporting information can be downloaded at https://www.mdpi.com/article/10.3390/cancers18050875/s1: Figure S1: Genetic alteration profiles of genes associated with immune checkpoint inhibitor response in the cBioPortal cohort.; Table S1: Spreadheet of all synthetic lethal pairs; Table S2: Co-occurrence and mutual exclusivity analysis of location specific genes (Gene A) against all 35 genes from chi-square test (Gene B). Table S3: TIMER2.0 analysis showing correlation of gene expression and immune cell infiltration of bladder tumors.
