# Supplementary material for: Role of Spatial Heterogeneity in Muscle-Invasive Bladder Cancer on Overall Survival and Immunotherapy Response
Source: Cancers (Basel). 2026 Mar 9;18(5):875. doi: 10.3390/cancers18050875 (PMC12984956; doi:10.3390/cancers18050875)
Supplement: Supplementary file 1 [file cancers-18-00875-s001.zip › Supplementary table 2-3 figure S1.pdf]

Supplementary Table S2. Co-occurrence and mutual exclusivity analysis of location specific genes (Gene A) against all 35 genes from chi-square test (Gene B). These analyses were conducted stratified by location in addition to location agnostic.

Location	Gene A	Gene B	Log2 Odds Ratio	p-Value	Tendency
Dome	RYR1	SI	>3	0.006	Co-occurrence
Dome	RYR1	MCM3AP	>3	0.009	Co-occurrence
Dome	RYR1	PTPRT	>3	0.016	Co-occurrence
Dome	RYR1	KMT2B	>3	0.042	Co-occurrence
Dome	RYR1	FCGBP	>3	0.047	Co-occurrence
Dome	OBSCN	PIK3CA	>3	0.002	Co-occurrence
Dome	OBSCN	DNAH11	>3	0.002	Co-occurrence
Dome	OBSCN	HMCN1	>3	0.014	Co-occurrence
Dome	OBSCN	XIRP2	>3	0.025	Co-occurrence
Dome	OBSCN	LRRCC1	>3	0.039	Co-occurrence
Dome	OBSCN	EP300	2.915	0.042	Co-occurrence
All	RYR1	FCGBP	>3	<0.001	Co-occurrence
All	OBSCN	ASH1L	2.16	<0.001	Co-occurrence
All	BPTF	LRRCC1	2.03	<0.001	Co-occurrence
All	RYR1	KMT2B	2.02	<0.001	Co-occurrence

Supplementary Table S3. TIMER2.0 analysis showing correlation of gene expression and immune cell infiltration of bladder tumors.

Immune Cell type	CDKN2A		SPTAN1		BIRC6	
	Rho	p-value	Rho	p-value	Rho	p-value
**CD8+**	**0.259**	**< 0.001**	**0.123**	**0.018**	**0.13**	**0.013**
**CD4+**	-0.014	0.789	0.091	0.082	**-0.119**	**0.022**
**Treg ** **	-0.019	0.714	-0.058	0.268	-0.085	0.102
**B cell**	0.03	0.573	0.037	0.481	0.043	0.408
**Neutrophil**	**0.136**	**0.009**	0.052	0.324	**0.267**	**< 0.001**
**Monocyte ** **	**-0.147**	**0.005**	-0.044	0.400	-0.022	0.681
**Macrophage * **	-0.037	0.475	0.073	0.165	**0.264**	**< 0.001**
**Dendritic Cell**	**0.219**	**< 0.001**	**0.268**	**< 0.001**	0.027	0.609
**Natural Killer * **	**0.251**	**< 0.001**	**0.203**	**< 0.001**	**-0.265**	**< 0.001**
**Mast Cell *** **	-0.056	0.283	-0.022	0.680	**0.442**	**< 0.001**
**Cancer Associated fibroblast * **	0.048	0.360	**0.178**	**< 0.001**	0.097	0.064
**Common lymphoid progenitor *** **	0.099	0.058	**-0.139**	**0.008**	**0.274**	**< 0.001**
**Common myeloid progenitor *** **	0.081	0.122	0.091	0.080	0.097	0.064
**Endothelial cell * **	-0.068	0.195	0.066	0.208	0.036	0.489
**Eosinophil ** **	-0.068	0.195	**-0.089**	**0.088**	0.009	0.856
**Granulocyte-monocyte progenitor *** **	-0.068	0.195	0.095	0.070	0.076	0.145
**Hematopoietic stem cell *** **	**-0.117**	**0.025**	0.002	0.968	**0.274**	**< 0.001**
**T cell follicular helper ** **	-0.005	0.918	-0.063	0.227	-0.056	0.280
**T cell gamma delta ** **	0.058	0.265	-0.015	0.769	**-0.127**	**0.015**
**T cell NK *** **	-0.045	0.387	**0.133**	**< 0.001**	**-0.352**	**< 0.001**
**Myeloid derived suppressor cells **** **	**0.179**	**< 0.001**	**0.139**	**0.007**	0.093	0.073

* = EPIC; ** = CIBERSORT; *** = XCELL; **** = TIDE

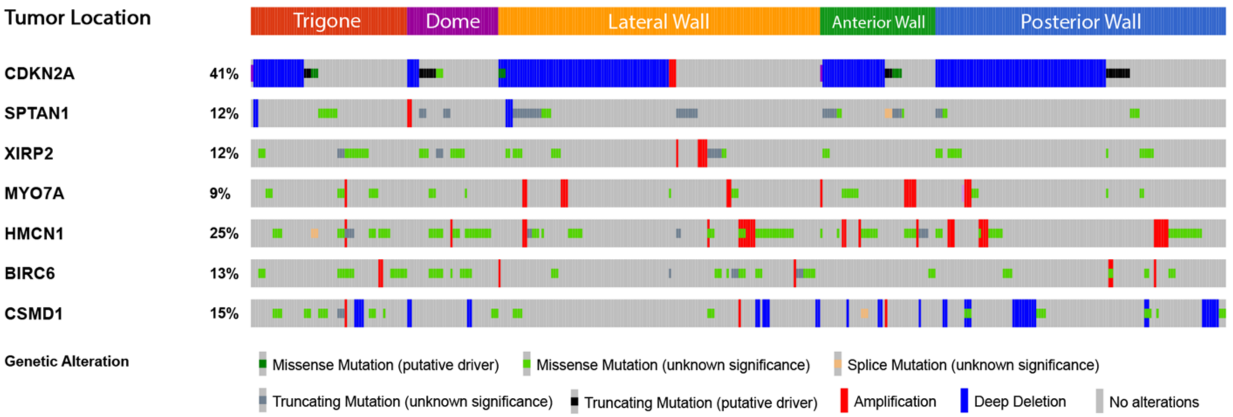

		CDKN2A	SPTAN1	XIRP2	MYO7A	HMCN1	BIRC6	CSMD1
**Total Sample Count**		570	570	570	570	570	570	570
**Genomic Alteration Frequency:** (mutations + CN variations)		43%	25%	16%	10%	28%	18%	16%
**Mutations**	**Mutation Frequency**	8.60%	23.30%	15.30%	7.20%	23.20%	17.20%	7.90%
	**Missense:** Benign or ambiguous	1.23%	6.84%	4.56%	2.28%	16.14%	8.77%	5.62%
	**Missense:** Pathogenic	2.28%	8.95%	0.00%	4.74%	3.86%	7.19%	1.23%
	**Missense:** Unknown Significance	0.53%	0.00%	8.60%	0.00%	0.53%	0.00%	0.00%
	**Truncating**	4.04%	7.02%	2.11%	0.00%	2.11%	1.23%	0.53%
	**Splice**	0.00%	0.53%	0.00%	0.00%	0.53%	0.00%	0.53%
	**Fusion**	0.53%	0.00%	0.00%	0.18%	0.00%	0.00%	0.00%
**Copy Number Variations**	**CN Variation frequency**	34%	1%	1%	3%	5%	1%	8%
	**Deep deletion**	33.68%	0.88%	0.00%	0.00%	0.00%	0.00%	7.54%
	**Amplification**	0.53%	0.35%	0.88%	2.98%	5.09%	1.23%	0.53%
**Gene expression levels that Immunotherapy responders are associated with (from ROCplot)**		Increased	Increased	Decreased	Increased	Decreased	Decreased	Decreased

Supplementary Figure S1: Genetic alteration profiles of genes associated with immune checkpoint inhibitor response in the cBioPortal cohort. The upper panel shows an OncoPrint visualization of individual cases across different tumor locations, with each row representing a gene and each column representing a patient sample. The lower panel provides a quantitative breakdown of genetic alterations. These seven genes were selected based on their significant association with ICI response in ROC Plotter analysis. Missense mutations were classified using AlphaMissense into benign/ambiguous, pathogenic, or unknown significance categories. The bottom row indicates whether increased or decreased expression of each gene was associated with ICI response in the ROC Plotter cohort.
